# Effects of caffeine ingestion on physiological indexes of human neuromuscular fatigue: A systematic review and meta‐analysis

**DOI:** 10.1002/brb3.2529

**Published:** 2022-03-23

**Authors:** Ruishan Sun, Junya Sun, Jingqiang Li, Shuwen Li

**Affiliations:** ^1^ College of Safety Science and Engineering Civil Aviation University of China Tianjin China

**Keywords:** caffeine, data synthesis, neuromuscular fatigue, physiological indexes

## Abstract

**Background:**

Caffeine is often used as a stimulant during fatigue, but the standard of characteristic physiological indicators of the effect of caffeine on neuromuscular fatigue has not been unified. The purpose of this systematic review and meta‐analysis is to summarize current experimental findings on the effects of caffeine on physiological indexes before and after neuromuscular fatigue, identify some characteristic neuromuscular physiological indexes to assess the potential effects of caffeine.

**Methods:**

The Preferred Reporting Items for Systematic Reviews and Meta‐analyses are followed. We systematically searched PubMed, Google academic, and Web of Science for randomized controlled trials. We searched for studies on caffeine's (i) effects on neuromuscular fatigue and (ii) the influence of physiological indexes changes. Meta‐analysis was performed for standardized mean differences (SMD) between caffeine and placebo trials in individual studies.

**Results:**

The meta‐analysis indicated that caffeine significantly improves voluntary activation (VA) (SMD = 1.46;95%CI:0.13, 2.79; *p* < .00001), PTw (SMD = 1.11, 95%CI: –1.61, 3.84; *p* < .00001), and M‐wave (SMD = 1.10, 95%CI: –0.21, 2.41; *p* < .00001), and a significant difference (*p* = .003) on measures of Peak Power (PP), and insignificant difference on measures of heart rate (HR) (*I* ^2 ^= 0.0, *p* = .84) and Maximal oxygen uptake (VO_2_) (*I* ^2 ^= 0.0, *p* = .76).

**Conclusion:**

The analysis showed that caffeine intake had a relatively large effect on VA, potentiated twitch (PTw), M‐wave, which can be used as characteristic indexes of caffeine's impact on neuromuscular fatigue. This conclusion tends to indicate the effects of caffeine on neuromuscular fatigue during endurance running or jumping or muscle bending and stretching. The caffeine intake had a big effect on the electromyogram (EMG) and peak power (PP), and its effect role needs to be further verified, this conclusion tends to indicate the effect of caffeine on neuromuscular fatigue during jumping or elbow bending moment movements. HR, VO_2_, maximal voluntary contraction (MVC) cannot be used as the characteristic indexes of caffeine on neuromuscular fatigue. This conclusion tends to indicate the effect of caffeine on neuromuscular fatigue during endurance exercise. However, the results of meta‐analysis are based on limited evidence and research scale, as well as individual differences of participants and different physical tasks, so it is necessary to interpret the results of meta‐analysis cautiously. Therefore, future research needs to explore other physiological indicators and their indicative effects in order to determine effective and accurate characteristic indicators of caffeine on neuromuscular fatigue.

## Graphical abstract

To summarize current experimental findings on the effects of caffeine on physiological indexes before and after neuromuscular fatigue; identifying some characteristic neuromuscular physiological indexes to assess the potential effects of caffeine.

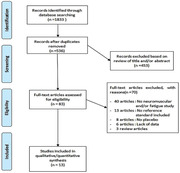

## 1 INTRODUCTION

Exercise‐induced fatigue can be defined as a transient decline in the neuromuscular system's ability to produce strength, which may be due to many changes along the path of movement. When the fatigue process stops in the central nervous system, it is called central fatigue. When the fatigue process stops at the neuromuscular junction or distal end, this is called peripheral fatigue (Amann, 2011; Gandevia, 2001). And caffeine also affects fatigue in different ways in different parts of the body. Caffeine is structurally similar to several endogenous metabolites and is distributed in intracellular fluids across the blood‐brain barrier and placenta (Arnaud, 1987); these properties allow caffeine to affect many human tissues, including the central nervous system, smooth muscle, cardiovascular system, and skeletal muscle (Thompson, 2002). In the sarcoplasmic reticulum, caffeine interacts with ryanodine receptors, caffeine may inhibit both adenosine receptors (Reinhold et al., 1989; Wada et al., 2013), and they are distributed in the nervous system, liver, heart, adipose tissue, and muscle (Reppert et al., 1991). Therefore, the effect of caffeine is a combination of effects on various tissues (Bazzucchi et al., 2011). Because of the effects of caffeine on different loci (i.e., central nervous system, Na^+^ /K^+^ ATPase activity, adenosine receptors, intracellular calcium, and/or plasma catecholamine concentration; Bishop, 2010), caffeine may be an effective ergogenic aid to counteract a decrease in exercise performance throughout the day.

Based on the above research progress, many scholars have carried out researches on the potential relationship between caffeine and neuromuscular fatigue (Connell et al., 2016; Duncan et al., 2019; Michael et al., 2013; Stadheim et al., 2014). For example, in a recent study, Bowtell et al. (2018) found that the improvement of the stretching performance of one knee after caffeine intake was related to the improvement of central and peripheral fatigue index. Studies have also been conducted on the central and peripheral responses to caffeine consumption during muscular endurance performance; this provides insight into the importance of caffeine's effects on neuromuscular fatigue. Caffeine inhibits the A1 adenosine receptor and the postsynaptic A2a receptor in the central nervous system (Davis et al., 2003; Jayne & Kalmar, 2005) and muscles (Bazzucchi et al., 2011; Tarnopolsky & Cupido, 2000), thus improving the excitability in the spinal cord and vertebrae during movement and changing the activation of the cortex and muscles. And Bazzucchi et al. (2011) found that caffeine increases electromyography of the biceps (EMG) and the maximum constant force to bend the elbow joint at different angular velocities; caffeine may also increase neuromuscular efficiency, as caffeine consumption increases the conduction speed of muscle fibers. But there is also literature reported that decaf and placebo are not associated with neuromuscular health (Apostolidis et al., 2020), and whether caffeine intake has different effects on neuromuscular fatigue at the end of exercise in low‐ and high‐performing individuals is an unexplored question; the underlying mechanism of caffeine intake for neuromuscular or muscular endurance exercise performance is also controversial.

Therefore, studies have explored the potential effects of caffeine on neuromuscular fatigue through changes in physiological indicators. For example, Santos et al. (2020) used the changes of postexercise voluntary activation (VA) and enhanced 1 Hz force twitched (TW) as markers of neuromuscular fatigue to explore the influence of exercise level on caffeine‐induced neuromuscular fatigue. Franco‐Alvarenga et al. (2019) validated whether the increase in endurance performance after caffeine intake coincided with changes in primary motor cortex (MC) and prefrontal cortex (PFC) activation, neuromuscular efficiency (NME), and electroencephalogram (EEG). And maximum voluntary traction (MVC) is an indicator of global fatigue; central fatigue is measured by autonomic activation (VA) through twitch interpolation technology, and twitch torque (an indicator of peripheral fatigue) (Kevin et al., 2018; Prasartwuth et al., 2005; Racinais et al., 2007; Takashi et al., 2005).

Although there have been some meta‐analyses of caffeine's effects on muscle strength and physiological performance (Diego et al., 2017; Jozo et al., 2018), but to our knowledge, to date, there is no systematic literature review or meta‐analysis of the effects of caffeine intake on a variety of physiological indicators. Thus, the purpose of this study was to systematically review the literature on the effects of preexercise caffeine intake on multiple physiological indicators before and after neuromuscular fatigue, and a meta‐analysis is then used to identify the characteristic indexes of the effects of caffeine on neuromuscular fatigue.

## 2 METHODS

### 2.1 Search strategy and selection criteria

This systematic review and meta‐analysis are reported in accordance with the Preferred Reporting Items for Systematic Reviews and Meta‐Analyses (PRISMA) statement (Ziegler et al., 2011). And all search strategies and methods were determined before the onset of the study.

We selected relevant studies published between January 1, 1950, and July 20, 2020, by searching PubMed, Scopus, Google Academic, Web of Science, and Networked Digital Library of Theses and Dissertations. We applied no language restrictions. The search terms were “caffeine” AND (“neuromuscular fatigue” OR “physiological” OR “indexes” OR “effect”).Other search terms, such as “neuromuscular” AND (“function” OR “performance” OR “fatigue” OR “power”) and “physiological indexes” AND (“voluntary activation” OR “Twitch” OR “M‐wave” OR “electromyogram” OR “maximal voluntary contraction”). We considered all potentially eligible studies for review, Inclusion criteria specified that studies were published in English or were available in translation to English. We also did a manual search, using the reference lists of key articles published in English. When possible, these were combined with a sensitive search strategy to identify trials performed in “humans.”

The inclusion criteria were as follows: (1) employed the randomized, double‐blind, crossover design; (2) subjects characteristics without chronic fatigue syndrome or disease; (3) when caffeine and placebo were ingested, there is no other potentially ergogenic compounds interference; (4) the selected studies were published in journal, or a doctoral or a master's thesis, with an experimental trial; (5) exercise/performance test includes neuromuscular fatigue or its manifestations. The following were disregarded: (1) any of the above criteria were violated; (2) review articles or case reports; (3) the article did not include an analysis of neuromuscular fatigue or functional studies; (4) the study with samples that had physical problem or bad diets habits; (5) articles with animal studies; and (6) more than 25% subjects’ dropouts.

### 2.2 Study selection and data extraction

**FIGURE 1:**
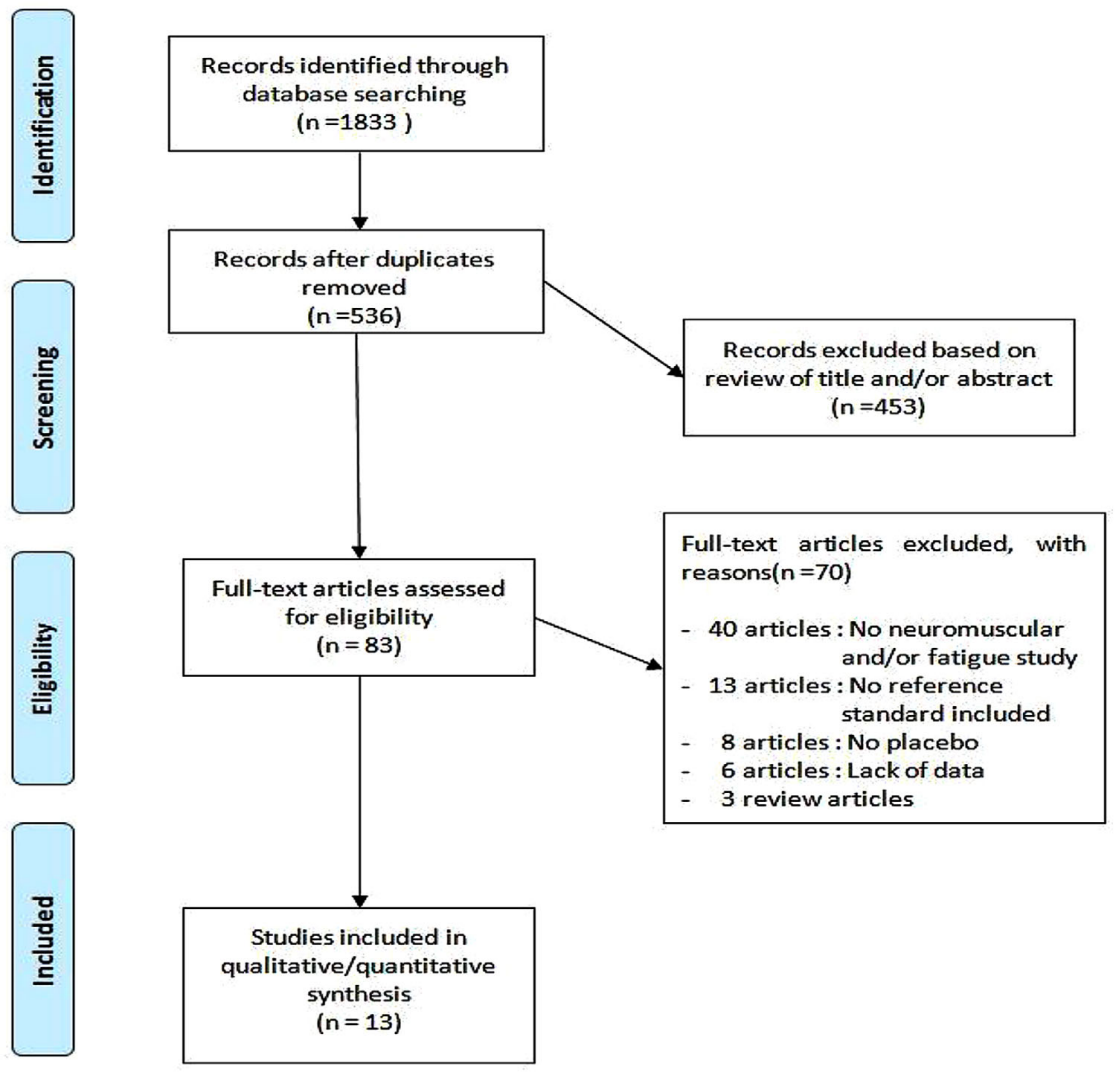
Flow diagram of the search and study selection process

The search in the databases provided 1833 articles. After 1297 duplicates were removed, all titles or abstracts were reviewed for relevance. Of the articles found, 453 were excluded after reading the title and abstract, resulting in 83 articles of which the full text was read. Of these 83 articles, 70 were excluded for the following reasons: no neuromuscular and/or fatigue study, no reference standard included, no placebo, lack of data, belong to review articles. The flow diagram of the search and study selection process are depicted in Figure 1. All the 13 literatures included the neuromuscular fatigue and physiological indicators of caffeine.

The methodological quality of studies was assessed using the PEDro scale, which has been reported to be valid and reliable (Maher et al., 2003; Natalie & Morton, 2009). The authors of the article assessed the selected studies, independent of each other. Any observed differences were resolved through discussion and consensus.

We extracted the general information of the trials from selected study. Subjects characteristics included in the analysis age, habitual caffeine intake of the participants, the amounts caffeine and placebo, time of ingestion before the experimental session(s), exercise/performance test and the PEDro Scale of study quality, and the basic features of the each selected study are shown in Table 1. The outcome measures and events were independently extracted by authors using a spreadsheet (Microsoft Excel 2010, USA), web plot Digitizer (WPD), and Engauge Digitizer (ED). Several studies included measurements in different types of performance outcomes, or even different types of tests for the same performance outcome. In these cases, each test was treated as a single and independent set of data for the meta‐analysis and included in the appropriate performance outcome.

**TABLE 1:** General characteristics of the studies included

Study	Study design	Subjects’ characteristics	Subjects age (years)	Habitual caffeine ingestion	Caffeine dosage	Time of ingestion before the experimental session(s)	Exercise/performance test	physiological index	PEDro scale
Santos et al. 2020	Double‐blind, crossover	19 male cyclists, body mass: 73.5 ± 8.7 kg, body fat: 10.5± 4.9%	33.5 ± 5.2	NQ	5 mg kg^−1^	60 min	4‐km cycling TT	VA/HR/VO2/MVC/PTw/M‐wave	10
Baz Zucchi et al. 2011	Double‐blind	14 moderately active male, body mass 72.3 ±5.6 kg	23.8 ± 2.8	<200 mg per week	6 mg kg^−1^	60 min	Elbow flexion torque	EMG_RMS_	9
Dittrich et al. 2019	Double‐blind, crossover	12 male runners (at least 3 years of experience) , body fat 9.4 ± 2.7%	31.3 ± 6.4	mean 105 ± 82 mg, range 10−235 mg for day	300 mg	45–60 min	Run a longer distance	VA/HR/VO2	10
Kalmar and Cafarelli 1999	Double‐blind	11 male, weight 71.4 ± 9.0 kg	22.3 ± 2.4	<200 mg per week	6 mg kg^−1^	60 min	Isometric torque of the quadriceps femoris, isometric knee extension	VA/MVC	8
Peltier et al. 2011	Double‐blind, crossover	13 trained adult males, body mass 71.7 ± 5.1 kg, Body mass index 22.4 ± 2.1 kg.m^−2^, Body Fat 14.0 ± 3.3 %	29.6 ± 9.2	1‐ 2 cups per day	150 mg	15 min	2 h treadmill run	VA/MVC	10
Marina et al., 2018	Double‐blind, crossover	14 male judo athletes (practice 12.9 ± 6.4), body mass 76.6 ± 12.7 kg, body fat of 12.9 ± 9.9 %	22.5 ± 7.1	moderate quantities of coffee during the day.	5 mg kg^−1^	60 min	The vertical jump, handgrip maximal strength, judogi grip strength	PP	10
San Juan et al. 2019	Double‐blind, crossover	8 male athletes, body‐mass: 65.63 ± 10.79 kg, Body Mass Index (BMI): 22.69 ± 1.31, load Wingate test: 4.91 ± 0.82	22.0 ± 1.778	NQ	6 mg kg^−1^	30 min	Handgrip and countermovement jump	PP	9
Mesquita et al., 2020	Double‐blind, crossover	18 male, body mass: 75.3 ± 7.4 kg	26.6 ± 3.1	average daily of 184 ± 157 mg	6 mg kg^−1^	60 min	Series of 40 jumps until task failure	VA/MVC/PTw/M‐wave	10
Apostolidis et al., 2020	Double‐blind, crossover	20 male soccer players, body mass 74.16 ± 7.52 kg, body fat 11.46 ± 3.25%	23.5 ± 3.5	Average daily of 85 mg	6 mg kg^−1^	60 min	Countermovement Jump and running	HR	10
Santos‐Mariano et al., 2019	Double‐blind, crossover	11 well‐trained young males’ sprinters and jumpers, weight 69.9 ± 6.4 kg	18.7 ± 2.7	<80 mg d^−1^	5 mg kg^−1^	50/75 min	Countermovement jump, sprint training and resistance training	VA/MVC/PTw/M‐wave	10
Black et al., 2015	Double‐blind, crossover	12 (6 men and 6 women)	college‐age	<40 mg d^−1^	5 mg kg^−1^	60 min	30 min of submaximal leg or arm cycling followed by a 10‐min time‐trial performance	MVC/HR/VO2	9
Bowtell et al., 2018	Double‐blind	9 male recreational athletes, weight: 78.4 ± 2.2 kg	26.0 ± 2.7	no‐habitual caffeine consumers	6 mg kg^−1^	60 min	Intense single‐leg knee extensor exercise to task failure	PTw/M‐wave	9
Fimland et al., 2010	Double‐blind, crossover	13 male, weight 76 ± 6 kg	23 ± 3	145 ± 88 mg/week	6 mg kg^−1^	60 min	Brief plantar flexion maximum voluntary isometric contractions	EMG_RMS_/M‐wave	10

NQ, no questionnaire survey.

### 2.3 Statistical analysis

The meta‐analysis was performed using STATA (version 15.1). Descriptive analysis was performed using Excel 2010, descriptive data of the caffeine and placebo groups are reported as mean ± standard deviation (SD). The *I* ^2^ statistic and 95%confidence intervals (95%CI) were used to assess the level of heterogeneity in the included sample. The *I* ^2^ statistic with values from < 50% represents small amounts of inconsistency, 50–75% represents moderate heterogeneity, and >75% represents high level of heterogeneity. The statistically significant differences threshold was set a priori at *p* < .05. The publication bias on the meta‐analysis was addressed by the Trim‐and‐Fill method (Duval & Richard, 2000). And the SMDs of >0.8, 0.5–0.8, 0.2–0.5, and ≤0.2 were considered to represent very large, large, medium, small effects, respectively (Christopher, 2019).

In the forest plot drawn by Revman software in this paper, when most of the prismatic combination effect size indicator patterns of a study fall on the right side of the invalid line, it can be considered that the indicators have an influence on the change of the reflected result. When most of the prisms fall on the left side of the invalid line, it can be considered that the index has no influence on the change of the reflected result.

The data of neuromuscular physiological indexes were collected according to change from pretest to posttest; statistical analysis was conducted on the (post–pre) /pre × 100% (Sébastien et al, 2011). Subgroup analyses for the effects of caffeine on physiological indexes of human neuromuscular fatigue were performed for the following study characteristics: (1) voluntary activation (VA) (the VA was calculated by the interpolated‐twitch technique) (Merton, 1954); (2) heart rate (HR) (HR refers to the number of heart beats of the human body, usually measured by an electrical monitor); (3) potentiated twitch (PTw) (it's neuromuscular pumping, stimulating with an electrical stimulator, recording the twitch of the muscle); (4) M‐wave (it's a waveform in an ELECTRO‐cardiogram; (5) maximal voluntary contraction (MVC) (a weight that can be lifted only once during strength training; e.g., using barbells); (6) maximal oxygen uptake (VO_2_) (an important indicator of aerobic capacity of the human body, usually analyzed with a gas analyzer); (7) electromyogram (EMG) (EMG refers to the analysis of muscle load recorded by electromyography biograph); (8) peak power (PP) (namely the time that power of a peak value to next peak value power; peak value is the wave peak that shows heart rate skin electricity or muscle electricity).

## 3 RESULTS

Table 1 depicts the general data of the studies included in this review. All of the studies used a double‐blind design. The 13 studies published between 1999 and 2020. A total of 198 participants were involved, with an average of 13 participants per article; one study has a minimum of 8 participants (San et al., 2019). Eight studies used samples of healthy athletes or trained person; five studies used healthy, untrained people. In 13 studies, body weight ranged from a mean of 65.6–78.4 kg. The mean age was between 18 and 35 years in 13 studies; college‐age also falls into this category. Eight studies reported habitual caffeine ingestion, with reported a small range of habitual ingests among the subjects (0–184 mg kg^−1^ per day). Seven studies reported the caffeine dosage was 6 mg kg^−1^, and four studies reported was 5 mg kg^−1^. Time of caffeine ingestion is between 45 min and 75 min before the onset of experimental measurements, but there were two studies reported the time were 15 min and 30 min, respectively. There are many forms of exercise/performance test. The PEDro methodological quality score ranged from 8 to 10; the mean score was 9.5. Only one study received the lowest score (PEDro score = 8); this lowest score was categorized as being of “good methodological quality,” so all studies were categorized as being of “good/excellent methodological quality.” More detailed individual characteristics of the included studies are reported in Table 1.

**FIGURE 2:**
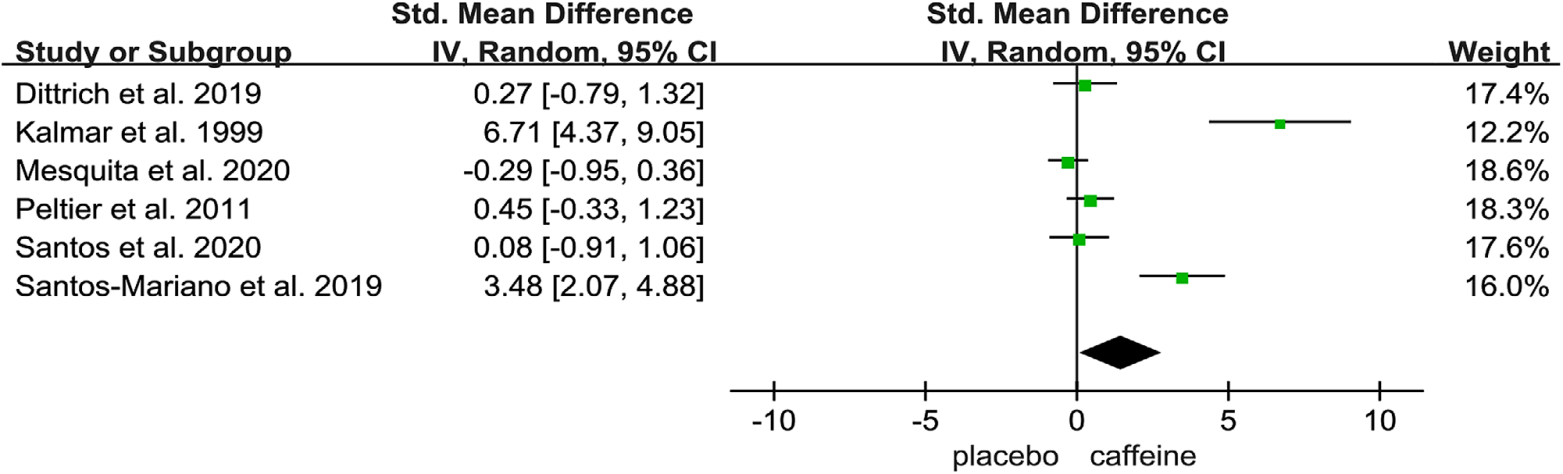
Effect of caffeine on voluntary activation (VA)

**FIGURE 3:**
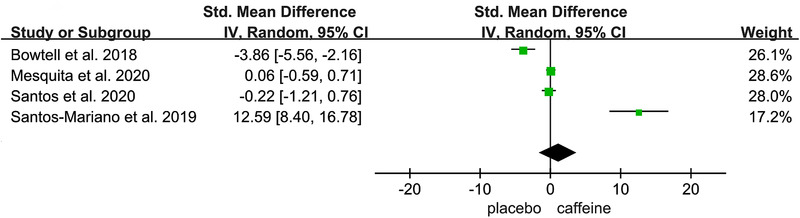
Effect of caffeine on Potentiated Twitch (PTw)

**FIGURE 4:**
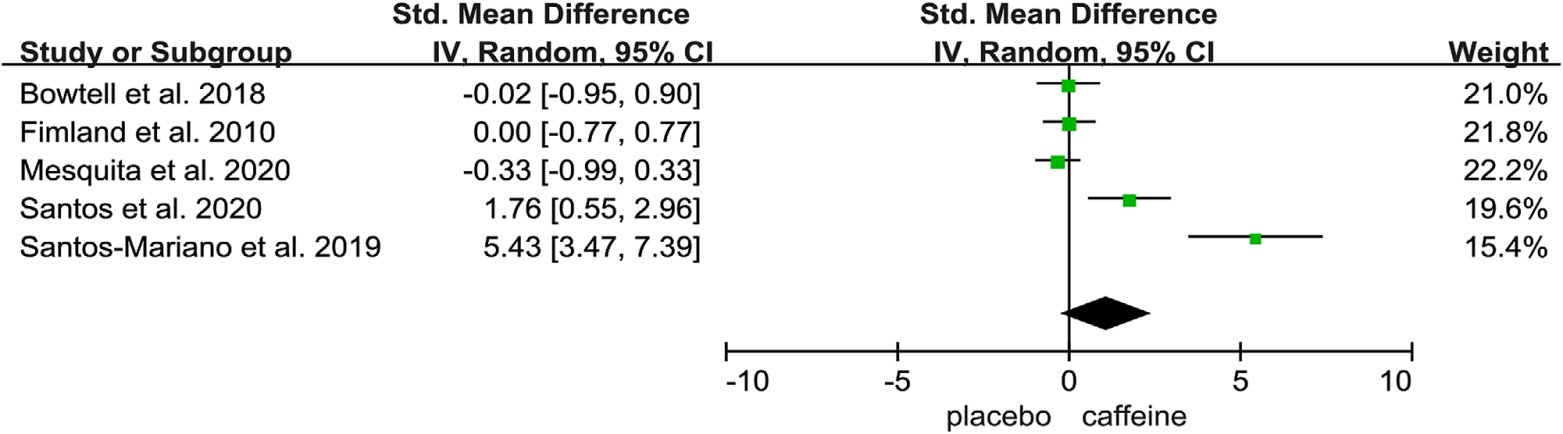
Effect of caffeine on M‐wave

**FIGURE 5:**
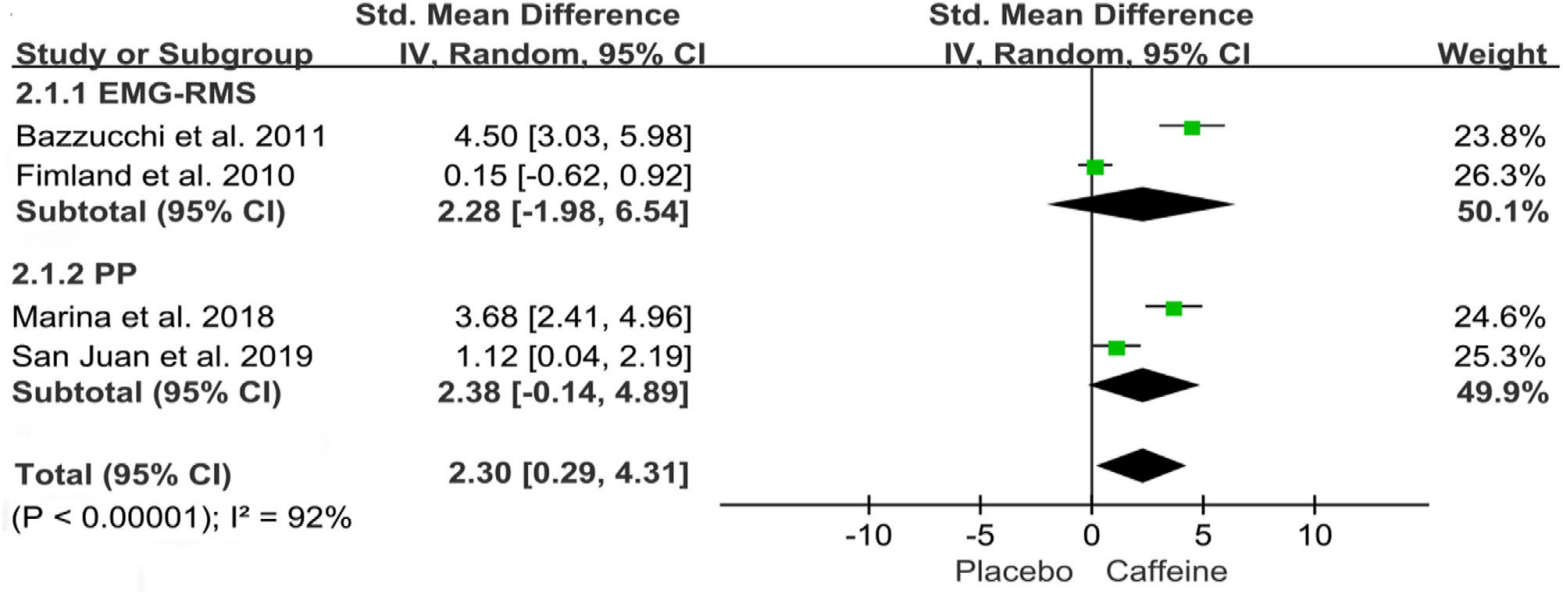
Effect of caffeine on electromyogram root mean square (EMG_RMS_), Peak Power (PP)

**FIGURE 6:**
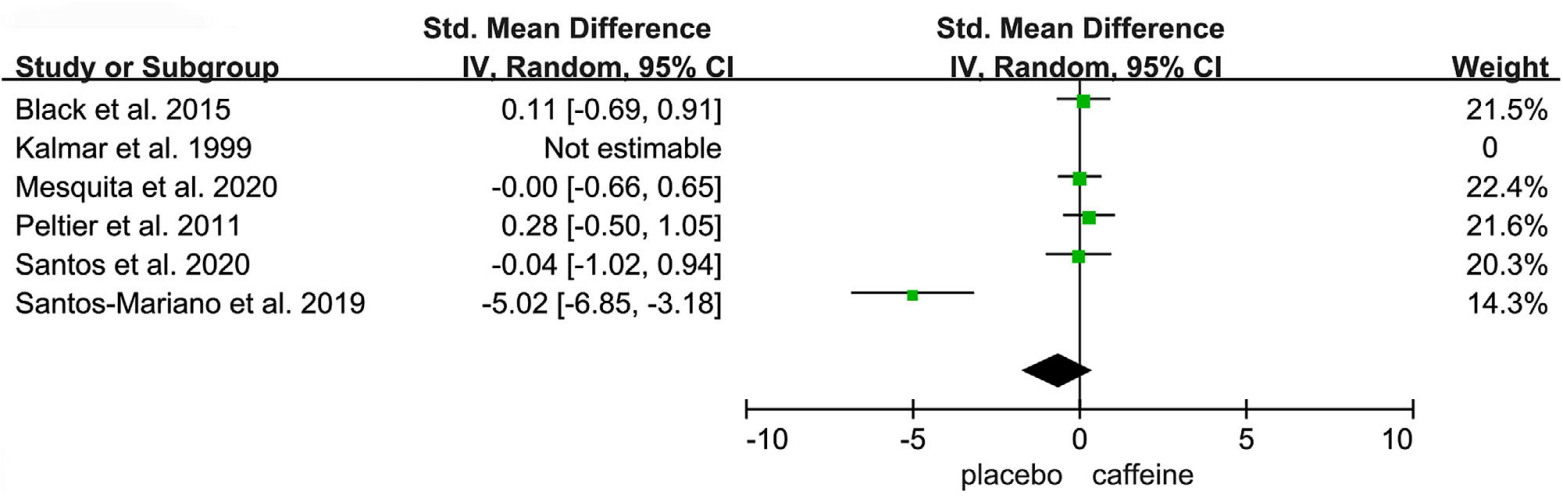
Effect of caffeine on maximal voluntary contraction (MVC)

Figures 2, 3, 4, 5, 6 show the effect of caffeine intake on physiological indexes (VA, PTw, M‐wave, EMG, and PP, MVC) of neuromuscular fatigue. And these figures show the comparisons between the trials with caffeine intake versus placebo trials for the different types of physiological indexes analyzed. The forest plots showed that caffeine intake had a relatively large effect on VA, PTw, and M‐wave (Figures 2, 3, 4); no effect on HR and VO_2_; and the EMG and PP indexes (Figure 5) had a big effect. However, due to the small number of included studies, its effect role needs to be further verified. Table 2 shows the results from all of the physiological index's analysis. Results of the meta‐analysis indicated a significant difference (*p* < .00001) between the caffeine and placebo trials on measures of indexes, and a significant difference (*p* = .003) on measures of PP, and insignificant difference on measures of HR (*p* = .84) and VO_2_ (*p* = .76). The meta‐analysis indicated that caffeine significantly improves VA (SMD = 1.46; 95%CI: 0.13, 2.79; *p* < .00001), PTw (SMD = 1.11, 95%CI: −1.61, 3.84; *p* < .00001), and M‐wave (SMD = 1.10, 95%CI: −0.21, 2.41; *p* < .00001). And the effects on other physiological indexes are shown in the Table 2. The *I* ^2^ statistic showed low heterogeneity for the studies assessing HR (*I* ^2 ^= 0.0, *p* = .84) and VO_2_ (*I* ^2 ^= 0.0, *p* = .76). The trim‐and‐fill method changed the SMD for HR from 0.41 (95% CI: 0.02, 0.8) to 0.46 (95% CI: −0.44, 3.36) and for VO_2_ from 0.14 (95% CI: −0.35, 0.63) to 0.09 (95% CI: −0.3, 0.48). The SMD, *I* ^2^, *p* value, and other statistic of meta‐analysis can be found in Table 2.

**TABLE 2:** Results from all of the physiological indexes meta‐analysis

Subgroup analysis	Number of trials	SMD [95%CI]	*I* ^2^ statistic (%)	*p* Value	Mean caffeine dose (mg/kg [range])
VA	68	1.46 [0.13, 2.79]	90	<.00001	5.1[3–6]
HR	52	0.41 [0.02, 0.8]	0	.84	5.5 [4.7–6]
PTw	46	1.11 [−1.61, 3.84]	94	<.00001	5.5 [5–6]
M‐wave	59	1.10 [−0.21, 2.41]	89	<.00001	5.6 [5–6]
MVC	73	−0.64 [−1.72, 0.44]	86	<.00001	5 [3–6]
VO_2_	32	0.14 [−0.35, 0.63]	0	.76	5.3 [3–6]
EMG_RMS_	27	2.28 [−1.98, 6.54]	96	<.00001	6 [6]
PP	22	2.38 [−0.14, 4.89]	89	.003	5.5 [5–6]

SMD, standardized mean difference; CI, confidence interval.

## 4 DISCUSSION

The results of the meta‐analysis were that, overall, caffeine can affect changes in certain physiological indexes during neuromuscular fatigue, but the effects of caffeine on physiological indexes were different. Compared with placebo, caffeine had a better effect on recovery of some physiological indexes, but no effect or even side effects on others. As for the current results, the effects of caffeine on body indicators are related to measurements in different parts of the body, as well as caffeine intake, time of day, different performance tests, number of studies, and journal in which the study was published. The previous meta‐analysis focused on the effects of caffeine on muscle strength, power, endurance, and physical performance (Polito et al., 2016), but with little paid to neuromuscular fatigue and no meta‐analysis of physiological indexes under neuromuscular fatigue conditions. Our novel results show that caffeine restores certain physiological indexes changes; in addition, the degree of neuromuscular fatigue can be predicted according to the changes of corresponding physiological indexes. Therefore, the effects of caffeine on physical indicators can be used to inform human neuromuscular fatigue, as well as the alleviate and restore potential of caffeine.

### 4.1 Voluntary activation (VA)

The VA and its changes were used as indices of central fatigue (Gandevia, 2001). Millet Guillaume and Lepers (2004) explained that at least part of the changes in VA after running exercise has been attributed to the inhibitory effect if afferent fibers. And in a previous study, energy drink has been observed to improve VA changes slightly (Millet et al., 2003). Our meta‐analysis indicated that compared with placebo, caffeine has a significant effect on VA, the reduction of VA indicator pre‐ and posttest is small, based on our results VA can be used as a physiological indicator of neuromuscular fatigue characteristics, and VA indexes are useful in indicating the potential effects of caffeine on neuromuscular fatigue recovery. Based on Table 1, this conclusion tends to indicate the effect of caffeine on neuromuscular fatigue during endurance running and other similar sports. This is consistent with what Kalmar and Cafarelli (1999) observed about an increase in maximal VA produced by the muscles after 6 mg kg of caffeine ingestion. Four studies (Dittrich et al., 2021; Mariano et al., 2019; Santos et al., 2020; Sébastien et al., 2011) recruited athletes as subjects, and these reported a significant effect in VA, and VA indicator was evaluated using only running and jump tests. These results are somewhat unexpected as other studies. For example, Mesquita et al. (2020) observed caffeine intake did not attenuate the significant decrease of VA throughout fatigue and recovery. Santos et al. (2020) also observed that regardless of the supplement, postexercise VA was lower in low‐performing participants than in high‐performing, and suggested that the central fatigue during a 4‐km cycling test is also affected by the participants’ performance levels. As depicted in Dittrich et al. (2021) research, VA was significantly decreased after the exercise, but it was not observed interaction between caffeine and placebo trials. And similar results have been found in recent studies (Ansdell et al., 2018; Bowtell et al., 2018; Camati et al., 2018; Konings et al., 2017). Thus, despite the caffeine benefits, it is still unclear how caffeine might affect the neuromuscular fatigue. So further research is needed to explore the VA can be used as one of the characteristic indexes to evaluate neuromuscular fatigue. Therefore, more experiments on VA indicator and relevant statistical induction are needed, especially the effect of caffeine on VA indicator under the condition of neuromuscular fatigue.

### 4.2 Potentiated twitch (PTw)

The PTw and the changes from baseline to postexercise in PTw were used as markers of peripheral fatigue (Amann, 2011; Santos et al., 2020). In peripheral fatigue, different training programs lead to inconsistency in PTw recovery time (24, 48 h or sometimes more than 4 days; Kevin et al., 2018; Prasartwuth et al., 2005; Racinais et al., 2007; TAKASHI et al., 2005). And PTw was significantly depressed during fatigue and recovery (Mesquita et al., 2020). In PTw testing result, Santos et al. (2020) observed that caffeine intake after exercise resulted in a slower reduction in PTw compared with placebo. And specifically in healthy male participants, the PTw amplitude induced by low‐frequency stimulation trains delivered to the common peroneal nerve during dorsiflexion was increased after caffeine ingestion (Bowtell et al., 2018). Our meta‐analysis observe that compared with placebo, PTw was significantly different before and after test when after caffeine ingestion, this is consistent with the above research conclusions. Based on Table 1, this conclusion tends to indicate the effect of caffeine on neuromuscular fatigue during leg movements such as jumping. However, Bowtell et al. (2018) observed that PTw amplitude elicited by peripheral nerve electrical in the resting muscle was not affected by caffeine or placebo ingestion, but PTw contraction time was significantly shorter in the caffeine trial. However, a small number of studies have shown that certain postexercise PTw is not affected by caffeine, as Santos‐Mariano et al. (2019) reported that PTw reduced after the half‐squat exercise but returned to baseline 24 h postexercise and was not affected by caffeine ingestion. So, the potential effect of caffeine on PTw in neuromuscular fatigue needs to be further explored, but most studies point that PTw can be used as one of the characteristic indexes to assess whether the neuromuscular has reached the fatigue status.

### 4.3 M‐wave

From Figure 4, our meta‐analysis shows that M‐wave has a significant difference between caffeine and placebo, and this phenomenon is consistent with current conclusions. Based on Table 1, this conclusion tends to indicate the effect of caffeine on neuromuscular fatigue during jumping or muscle flexing and stretching. Mesquita et al. (2020) found that the participants who benefited from an improved performance on the caffeine day had an accelerated postexercise recovery of M‐wave amplitude. Santos et al. (2020) observed that M‐wave increased from baseline to postexercise in the caffeine condition, and postexercise values for the caffeine condition are higher than postexercise values for the placebo condition. And a caffeine‐induced increased in M‐wave has been reported (Camati et al., 2018; Froyd et al., 2016; Temesi et al., 2017) and this might be associated in extracellular K^+^ accumulation after a high‐intensity exercise, which may preserve muscle fiber membrane excitability (Mohr et al., 2011; Simmonds et al., 2010). Similar explanations have been supported in other studies. Shushakov et al. (2010) observed an inverse relationship between M‐wave area and venous potassium concentration during fatiguing static and dynamic exercise. And caffeine prevented the reduction in M‐wave amplitude and area that occurred after fatiguing exercise in the placebo trial (Bowtell et al., 2018). In fact, it has been demonstrated that the M‐wave can assess a caffeine‐induced attenuation of neuromuscular transmission failure after fatigue (Bowtell et al., 2018), caffeine regulates the recovery of M‐wave amplitude (Mesquita et al., 2020), and caffeine ingestion increases the maximal M‐wave amplitude in fresh muscle. So, M‐wave can serve as one of the characteristic physiological indexes of caffeine's potential effects on neuromuscular fatigue.

### 4.4 Electromyogram root mean square (EMG_RMS_) and peak power (PP)

#### 4.4.1 EMG_RMS_

Bazzucchi et al. (2011) observed that after caffeine ingestion, EMG_RMS_ was significantly increased relative to the PRE condition; in contrast, the EMG_RMS_ was similar between PRE and POST in the placebo trials, and the increase in the excitability of rapid exercise units was also consistent with the increase in EMG_RMS_ after caffeine intake, but not with placebo. And in the current study, normal EMG was used to study the regulation of the central nervous system after caffeine intake, and the results showed a significant decrease immediately after fatigue, usually gradually returning to the prefatigue values (Fimland et al., 2010). Our meta‐analysis result is same as the current study; compared to placebo, caffeine ingestion has a significant effect on EMG_RMS_ before and after neuromuscular fatigue. Based on Table 1, this conclusion tends to indicate the effect of caffeine on neuromuscular fatigue during exercises such as elbow bending moment.

#### 4.4.2 PP

The caffeine ingestion showed a significantly improved the PP and further increased response speed, thereby shortening the time to the PP (San et al., 2019). Our meta‐analysis observed that compared to placebo, caffeine had a significant effect on the PP indicator. It has the same result as that from the PP produced was extracted as an indicator of neuromuscular fatigue (Sébastien et al., 2011), and our result is consistent with one other meta‐analysis that have found similar result for the PP (Jozo et al., 2018). Based on Table 1, this conclusion tends to focus on the effect of indicating caffeine on neuromuscular fatigue during jumping exercise.

Although our meta‐analysis included few literatures on EMG_RMS_ and PP indexes, which may cause limitations, however combined with previous experimental conclusions, we can boldly point out that they do indicate the differential impact of caffeine on neuromuscular fatigue compared with placebo (EMG_RMS_ [SMD = 2.28, 95%CI: −1.98, 6.54; *p* < .00001], and PP [SMD = 2.38, 95%CI: −0.14, 4.89; *p* = .003]). So EMG_RMS_ and PP can be used as the characteristic physiological indexes to assess the potential effects of caffeine on neuromuscular fatigue.

### 4.5 Maximal voluntary contraction (MVC)

The MVC was assumed as a global indicator of fatigue (Kevin et al., 2018; Kevin et al., 2014; Prasartwuth et al., 2005), and it was also used for further statistical analysis of the neuromuscular function (Amann & Dempsey, 2008). But MVC remained depressed throughout the fatiguing activity and recovery, with no significant main effect of psychoactive drug or time × drug interaction (Kalmar & Cafarelli, 1999). And Mesquita et al. (2020) also found that caffeine did not affect MVC decline during fatigue, nor did it enhance fatigue recovery, and also did not diminish the central and peripheral fatigue shown throughout the fatigue regimen and the recovery process. Many studies also found that the MVC is not significantly altered by caffeine (Sébastien et al., 2011; Kalmar and, Cafarelli, 1999), and our meta‐analysis observed the same result. The reasons of this result are probably due to high interindividual changes (Sébastien et al., 2011), or due to a larger than expected day‐to‐day variation in MVC or fatigue from a previous testing day (Black et al., 2015). Therefore, the MVC can be used as an indicator of overall fatigue, but it cannot be used as a characteristic indicator of the effects of caffeine on neuromuscular fatigue. Based on Table 1, this conclusion tends to indicate the effects of caffeine on neuromuscular fatigue during endurance running or jumping.

### 4.6 Heart rate (HR) and oxygen uptake (VO_2_)

#### 4.6.1 HR

First, we performed a meta‐analysis of four selected studies on HR using a fixed effect model, the *p* value is .84, so there was no statistical significance between changes in HR and neuromuscular fatigue. Based on Table 1, this conclusion is biased to indicate the effect of caffeine on neuromuscular fatigue during endurance exercise. For example, HR is used to classify cardiopulmonary health, not neuromuscular. And for the cardiorespiratory fitness classification, HR was higher with caffeine than with placebo (Apostolidis et al., 2020). HR did not differ between the caffeine and placebo conditions for either leg cycling or arm crank cycling, and caffeine induced reductions in muscle pain and in the absence of changes in HR during submaximal exercise (Black et al., 2015). However, a significant main effect for time was found for HR during leg cycling, with HR increasing progressively over time compared to the first minute of the time trial (Black et al., 2015). This is because caffeine directly reduces parasympathetic nervous system activity, so caffeine ingestion during exercises HR response (Sondermeijer et al., 2002), but in higher intensity exercise, this difference tends to disappear because the sympathetic nervous system affects the automaticity of the sinoatrial node and has a major influence on HR control (Sonntag et al., 2012).

#### 4.6.2 VO_2_

The VO_2_ is same as HR; meta‐analysis uses the fixed‐effect models, that the three studies among VO_2_ is limited by small overall study size, there is no statistical significance between VO_2_ and neuromuscular fatigue (*p* = .76). At the same time, similar phenomena have been found in the literature that caffeine increased absolute and relative HR and VO_2_ compared to placebo (Santos et al., 2020). This may be related to the research emphasis of the included literatures; most of the studies used HR and VO_2_ as auxiliary physiological indexes of test and did not focus on the potential effects of caffeine. Thus, HR and VO_2_ are not characteristic indexes of neuromuscular fatigue. Based on Table 1, this conclusion is biased to indicate the effect of caffeine on neuromuscular fatigue during endurance exercise.

## 5 CONCLUSION

Caffeine has an impact on physiological indexes before and after neuromuscular fatigue, including VA, PTw, and M‐Wave, which can be used as characteristic indexes of caffeine's impact on neuromuscular fatigue; EMG and PP indexes, although few literatures were included, but combined with previous experimental conclusions, can also clearly indicate the potential effect of caffeine on neuromuscular fatigue. However, MVC indicator is only a physiological indicator of fatigue and cannot indicate the potential effect of caffeine on neuromuscular fatigue, so it cannot be used as a characteristic indicator of caffeine on neuromuscular fatigue, and according to our meta‐analysis and previous studies, HR and VO_2_ cannot be used as the characteristic indexes of caffeine's effect on neuromuscular fatigue. But the results of the meta‐analysis are based on limited evidence and studies size, and there are individual differences in participants, different physical tasks, so this meta‐analysis results need to be interpreted with caution. The physiological indicators screened with the effect of caffeine on neuromuscular fatigue indicators are limited by the different activity types of the subjects in the selected literatures; that is, the indicators of the selected characteristic physiological indicators are applicable to specific activity scenes.

In addition, neuromuscular fatigue is a comprehensive state of the human body. Therefore, the evaluation of neuromuscular fatigue needs to be indicated by multiple physiological indexes, and the potential effects of caffeine on neuromuscular fatigue also require multiple physiological indexes to explore. Therefore, future research needs to explore other physiological indexes and their indicative effects, to determine the effective and accurate characteristic indexes of caffeine's effect on neuromuscular fatigue. In the future, studies can be carried out to screen physiological indicators indicating the effects of caffeine on neuromuscular fatigue under certain activities, such as mental work, physical work, and a combination of mental and physical work.

## FUNDING

This work was supported by grants from the National Natural Science Foundation of China and Civil Aviation Administration of China (U1933122), the Scientific research Project of education Commission of Tianjin, China (2020KJ029).

## AUTHOR CONTRIBUTIONS

All the authors listed have made a substantial, direct, and intellectual contribution to the work, and approved it for publication. Ruishan Sun and Junya Sun contributed equally to this work and shall be considered as cofirst authors.

### PEER REVIEW

The peer review history for this article is available at https://publons.com/publon/10.1002/brb3.2529.
